# Meat inspection of reindeer – a rich source of data for monitoring food safety and animal and environmental health in Sweden

**DOI:** 10.1080/20008686.2017.1340695

**Published:** 2017-07-18

**Authors:** Arja Helena Kautto, Ivar Vågsholm, Rauni Niskanen

**Affiliations:** ^a^ National Food Agency, Food Control Division, Uppsala, Sweden; ^b^ Department of Biomedicine and Veterinary Public Health, Swedish University of Agriculture, Uppsala, Sweden; ^c^ Department of Microbiology, National Veterinary Institute, Uppsala, Sweden

**Keywords:** Meat inspection, reindeer, monitoring, food safety, animal welfare, environment

## Abstract

**Background**: ​This study scrutinized carcass conditions recorded in post mortem inspections (PMI) of reindeer (*Rangifer tarandus tarandus*, L.) during 2015–2016 because of the importance for monitoring food safety and animal and environmental health threats.

**Material and methods**: PMI results were retrieved from the National Food Agency. A negative binomial regression model was applied. For actual parameters, incident risk rate (IRR) with confidence intervals was calculated.

**Results and discussion**: The number of conditions found in PMI varied widely between years and batches. The most common conditions (43 and 57% of all reindeer slaughtered in 2015 and 2016, respectively) derived from non-zoonotic parasites as the most abundant one, *Hypoderma tarandi. Setaria* sp. as well as both inflammatory processes and trauma were found in low prevalences. Further investigation of interactions with slaughterhouse size and inspector experience is needed. The conditions found rarely indicated food safety hazards and no epizooties or zoonoses have been recorded in the past two decades. Visual PMI with complementary sampling for specific hazards in slaughterhouses could thus be a helpful tool for monitoring the health and welfare of the reindeer population, the food safety risks with reindeer meat, and the status of the environment.

​

## Background

**Figure 1.**
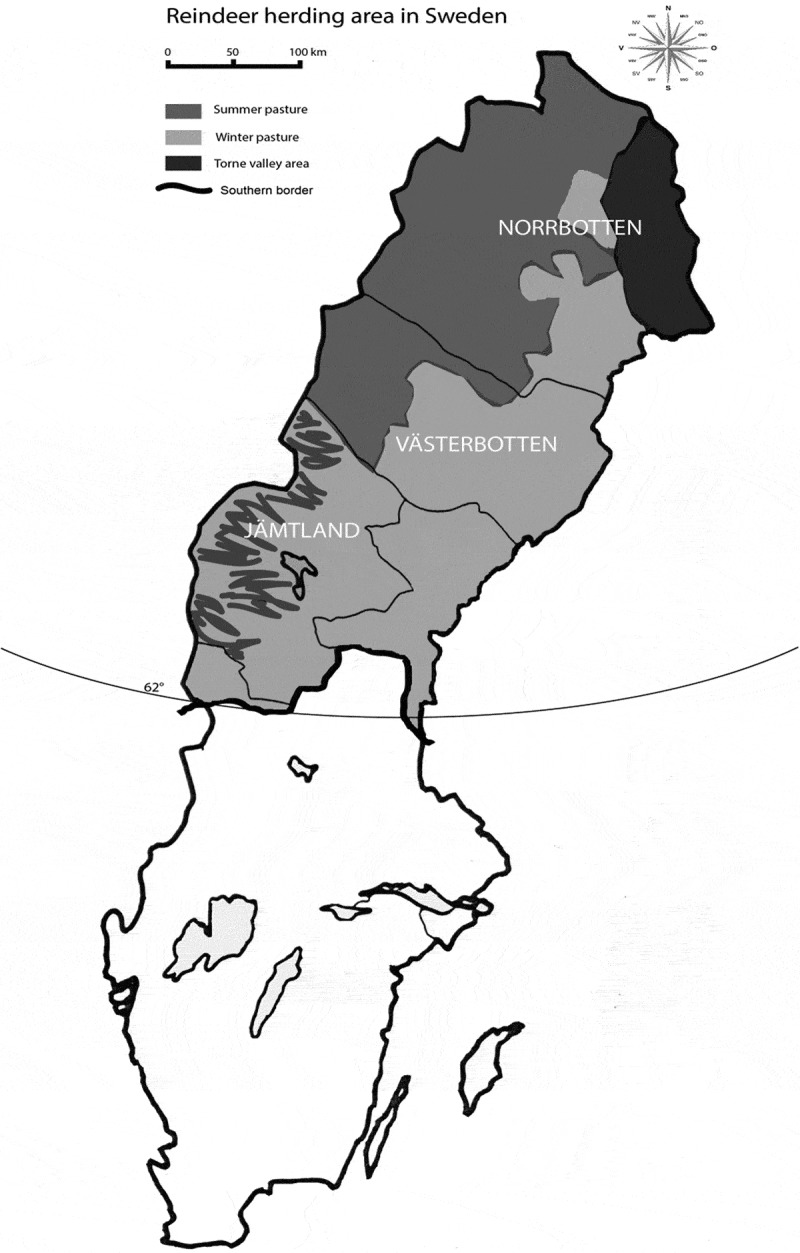
Swedish reindeer herding area.[5]​

Reindeer management has been an important part of the Sami people’s livelihood in Scandinavia since ad 800. Today, about 250,000 live reindeer (counted on 31 March annually [1]) graze freely across natural pastures in northern Sweden, with few human contacts. There are 51 Sami villages, with co-operative rights to herd reindeer on a large area in Northern Sweden, consisting of about 50% (200,000 km^2^) of Sweden’s total land area (Figure 1). Reindeer herding is an exclusive right of the indigenous Sami people with one exception, namely local farmers with a few reindeer each in the Torne valley along the Finnish border. Around 55,000 reindeer are slaughtered every winter (September–April) at reindeer slaughterhouses located in remote rural areas, with a three-week break during the reindeer rutting season in October.

Meat inspection (MI) focuses on any disease or condition that might affect public or animal health or compromise animal welfare.[2] MI of reindeer in slaughterhouses represents a census of the reindeer population going to slaughter, and thereby a rich source of useful information, for example about: (a) the health and welfare of the reindeer population; (b) the food safety risks entering the food chain with reindeer meat; and (c) the environment. This knowledge can help herders, authorities and others involved to optimise their activities and handle the risks. One environmental problem monitored in reindeer is caesium concentration, following the Chernobyl nuclear accident and its fallout on reindeer herding areas in Sweden. Reindeer can be considered a sentinel for environmental changes with possible consequences in a One Health perspective and slaughterhouses can act as useful hubs for monitoring and surveillance.

Compulsory MI of reindeer was implemented in Sweden in 1966.[3] Since 2006, MI has been performed according to the European Union (EU) Food Hygiene package (FHP),[2,4] in the same manner as MI at slaughter of sheep and goats. All reindeer must be slaughtered in approved slaughterhouses applying the same standards as for domesticated animals, and ante-mortem inspection is performed at the slaughterhouse within 24 hours before slaughter. Reindeer are gathered from the vast wilderness of northern Sweden, where weather conditions create logistical challenges, with associated high MI costs.[5] Official inspectors perform conventional MI on the slaughter line. Sampling for hazards not visible macroscopically is only performed if there are indications from food chain information (FCI). Sampling for residues of veterinary medicines, other undesirable substances (cadmium, lead, mercury) and pollutants (chlorinated organic hydrocarbons) is performed according to the national monitoring programme.[6]

This study focused on post mortem inspections (PMI), since findings in ante mortem inspections (AMI) are extremely rare (about 1 per 5000 reindeer and year). The aim of the study was to collate, scrutinise and discuss carcass conditions recorded in PMI of reindeer during 2015–2016, with a view to drawing inferences from the carcass conditions found and considering their usefulness for slaughterhouse monitoring of food safety, reindeer health and welfare and environmental health.

## Materials and methods

### Study design

Slaughter statistics, including documented carcass conditions, were retrieved from the National Food Agency (NFA) database. The official slaughter statistics 2015–2016 for reindeer were retrieved from Sami Parliament,[1] the national authority responsible for issues concerning the Sami minority in Sweden. Information concerning transport of reindeer to slaughter between Finland and Sweden was obtained from the Trade Control and Expert System (TRACES).[7]

Any carcass conditions found are recorded by inspectors on code level for every slaughter batch, not on individual reindeer level. Thus one reindeer can have more than one code registered, except for a few individual-specific codes. Every slaughter batch of reindeer is defined by its origin (mountain or forest Sami village), date, slaughterhouse and meat inspector (official veterinarian or auxiliary).

The inspectors who performed the MI were divided into two groups based on their experience. Members of the group with long experience (LE, six and seven individuals in 2015 and 2016, respectively) work almost daily with PMI of reindeer during the season, have been doing this work for more than three years and have inspected more than 10,000 reindeer. Members of the group with short experience (SE, 14 and 13 individuals in 2015 and 2016, respectively) are stand-in staff who work occasionally when needed, have been doing this for more or less than three years and have inspected fewer than 10,000 animals.

Slaughter dates in August–October were categorised as autumn slaughter, those in November–January as winter slaughter and those in February–May as spring slaughter. This categorisation was based on snow and weather conditions.

The analysis covered a total of five and four slaughterhouses considered large (annual kill >6000 reindeer) in 2015 and 2016, respectively, and six and eight slaughterhouses considered small (annual kill ≤6000 reindeer) in 2015 and 2016, respectively.

Origin of reindeer at slaughter was defined by FCI. Reindeer from mountain Sami villages (33 villages) were classified as mountain origin and reindeer from forest Sami villages (10 villages) and the Torne valley (eight villages) were classified as forest origin. Initially, data from all reindeer slaughtered were studied for prevalences. Slaughter batches coming from either Finland or Norway or of mixed origin prior to slaughter were excluded from further analyses.

The diagnostic groups of carcass conditions were based on the specific codes used in the NFA system (see Table 1). *Setaria* sp., a mosquito-borne filarioid nematode, was analysed separately. *Hypoderma tarandi* (reindeer warblefly), acute and chronic trauma were codes recorded only once per reindeer. *Hypoderma tarandi* is the most abundant parasite found, is easy to diagnose visually and can be related to both biology (weather conditions, type of ecosystem) and management (antiparasitic treatment, choice of grazing area). Acute trauma relates to animal welfare during handling and transport, and the degree of tameness of the reindeer. Chronic trauma may be linked to management failure but also many other causes, such as rutting fights, predator attacks or accidents (road and railroad). Inflammatory processes (IP) include a total of eight different types of inflammation with no aetiology defined. One reindeer can have more than one of these codes recorded and, because of this, within-batch variation and individual prevalence were not calculated.

**Table 1.** Diagnostic groups of registered conditions and diseases.

Group of carcass conditions	Includes	Comments
Parasites	Cysticercosis, onchocercosis, setariosis, dictyocaulus, elaphostongylosis, *Hypoderma tarandi, Dicrocoelium dendriticum*	Small abscesses and other damage in liver as traces after parasitism included here. Changes in lungs recorded here only when parasites visually confirmed. Cysticercosis laboratory confirmation demanded.
Acute traumatic lesions	Acute lesions	Acute: subcutaneous bleedings and fresh fractures in ribs or other bones, caused by humans or animals (e.g. goring) during handling of animals
Chronic traumatic lesions	Chronic lesions	Chronic: healed wounds and fractures after accidents on roads/railroads, rutting fights between males, predator attacks, even human-caused during management activities
Emaciation	Emaciation	Animals declared unfit for human consumption because of wasting. Absence of epicardial fat (serous atrophy around arteria coronaria), absence of abdominal fat tissue, gelatinous bone marrow. Muscle atrophy.
Inflammatory processes	Inflammatory processes	Peritonitis, pneumonia, pleuritis, pericardititis, perihepatitis, abscess, septicaemia, arthritis. Parasites not confirmed visually or in laboratory.
Poor slaughter hygiene	Carcass not clean	Faecal or other contamination on the carcass visually confirmed. Includes some cases after laboratory testing. Incomplete bleeding not included.
High caesium-137 and caesium-134 content	Content of Cs-137 and/or Cs-134 in carcass exceed 1500 Bq kg^–1^	Measurement done externally and confirmed at the laboratory. Bq = bequerel. Carcasses with levels of Cs exceeding 1500 Bq are declared unfit for human consumption.

### Statistical analyses

The selected models had to be parsimonious, simple, biologically plausible and capable of producing easily interpretable results. A significance level of *p* < 0.05 was set in statistical analyses.

Because of the strong overdispersion of the data, a negative binomial model was selected for the analysis.[8] The model run for parameters was weighted by batch size. Descriptive analysis was performed for each year separately. Analytical statistics were performed for both years separately and combined.

For all model runs, incident risk rate (IRR) with confidence intervals was calculated. All statistical analyses were performed using R software (version ‘Sincere Pumpkin Patch’, CRAN.R-project.com, 2016).

## Results

The number of reindeer slaughtered was 54,428 in 2015 and 55,535 in 2016. Nearly 70% of the slaughtered reindeer were calves.[1] Based on an average slaughter weight of 25 kg per reindeer,[1] production is 1375 t per year, which is equivalent to 5.5 kg/live reindeer in the winter herd. The slaughtered reindeer were almost exclusively from Sweden, with only about 5% each year coming from Finland to Swedish slaughterhouses near the eastern border and about 2% of Swedish reindeer being slaughtered in Finland.[7] Most of the slaughter (53%) took place in the northernmost county of the reindeer herding area (Norrbotten). The slaughter of reindeer was concentrated, with 50% of the slaughterhouses processing 80% and 85% of all reindeer in 2015 and 2016, respectively. There were 11 and 12 active slaughterhouses in 2015 and 2016, respectively.

Recorded conditions found most in PMI of all carcasses were parasites (43 and 57% in 2015 and 2016, respectively) followed by inflammatory processes (1.6 and 4.8% in 2015 and 2016, respectively), acute trauma (2.7 and 3.2% in 2015 and 2016, respectively), chronic trauma (0.6 and 1.0% in 2015 and 2016, respectively) and emaciation (0.3 and 0.2% in 2015 and 2016, respectively). Each carcass could have more than one condition recorded. Excessive amounts of caesium (Cs^137^/Cs^134^) were detected in 0.002% (one reindeer) and 0.008% (four reindeer) of cases in 2015 and 2016, respectively. Poor slaughter hygiene was recorded in 0.002% and 0.004% of cases in 2015 and 2016, respectively. No zoonoses or epizooties were observed within the study period. The filarioid nematode, *Setaria* sp., showed prevalence of 0.09% and 0.03% in 2015 and 2016, respectively, in both years in batches with origin in the Torne valley.

As the descriptive statistics show (Table 2), most of the reindeer slaughtered were of mountain origin. Batch size and number of conditions per batch varied widely in both years. *Hypoderma tarandi*, which is found subcutaneously on the back along and sagitally to the spine, showed higher batch and individual prevalence in 2016. Chronic trauma showed slightly higher individual prevalence in 2016. The batch prevalence of inflammatory processes was about 10% lower in 2016.

**Table 2.** Descriptive statistics for 2015 and 2016.

Covariate/factor	2015	2016
Number of slaughtered reindeer in analysis	49,966	52,635
Number of slaughtered reindeer of mountain origin	42,563	46,097
(% of total per year)	(85%)	(88%)
Number of slaughtered reindeer of forest origin	7,403	6,538
(% of total per year)	(15%)	(12%)
Number of batches (total in analysis)	327	389
Number of batches of mountain origin	240	279
(% of total per year)	(73%)	(72%)
Number of batches of forest origin	87	110
(% of total per year)	(27%)	(28%)
Batch size median	137	105
(min–max)	(4–525)	(2–458)
Batch size in large slaughterhouses,	128	113
median (min–max)	(4–525)	(2–458)
Batch size in small slaughterhouses,	132	103
median (min–max)	(4–355)	(7–395)
Total number of carcass conditions	39	50
recorded per batch median (min–max)	(1–705)	(1–654)
Number of batches with code *Hypoderma*	180	262
*tarandi* (batch prevalence %)	(55%)	(67%)
Within-batch prevalence *H. tarandi* amongst positive batches, median (min-max)	0.28 (0.003–1)	0.33 (0.004–1)
Total number of reindeer with condition	8,186	14,061
*H. tarandi* (individual prevalence %)	(16%)	(27%)
Number of batches with code acute trauma (batch prevalence %)	226 (69%)	268 (69%)
Within-batch prevalence acute trauma amongst positive batches, median (min–max)	0.02(0.003–0.51)	0.03(0.003–0.44)
Total number of reindeer with acute trauma (individual prevalence %)	1,285(2.6%)	1,463(2.8%)
Number of batches with code chronic trauma (batch prevalence %)	118 (36%)	143 (37%)
Within-batch prevalence chronic trauma amongst positive batches, median (min-max)	0.01(0.003–0.17)	0.02(0.002–0.24)
Total number of reindeer with chronic trauma (individual prevalence %)	289 (0.6%)	490 (0.9%)
Number of batches with at least one code in inflammatory processes (IP) (batch prevalence %)	198 (61%)	202 (52%)

Descriptive statistics for large and small slaughterhouses and inspector groups with long and short experience are presented in Table 3. Higher prevalences were recorded in small slaughterhouses and by inspectors with short experience for *Hypoderma tarandi* and acute trauma during 2015 and 2016, but for chronic trauma only during 2015 and for IP only during 2016. Lower prevalence was recorded for chronic trauma by inspectors with short experience during 2016. Higher prevalence of IP was recorded by inspectors with short experience during 2015.

**Table 3.** Descriptive statistics for large (>6000 annual kill) and small (≤6000 annual kill) slaughterhouses (SLH) and for inspector experience groups with long (LE) and short (SE) experience in PMI of reindeer for findings *Hypoderma tarandi* (*H. tarandi*), acute trauma, chronic trauma and inflammatory processes (IP) found in PMI of reindeer.​

Covariate/ factor	Large SLH		Large SLH		Small SLH		Small SLH		Inspector LE		Inspector LE		Inspector SE		Inspector SE	
**Year**	**2015**		**2016**		**2015**		**2016**		**2015**		**2016**		**2015**		**2016**	
	**No.**	**%**	**No.**	**%**	**No.**	**%**	**No.**	**%**	**No.**	**%**	**No.**	**%**	**No.**	**%**	**No.**	**%**
Slaughterhouse	5	45	4	33	6	55	8	67								
Inspector									6	30	7	35	14	70	13	65
Slaughtered	37,640	75	33,864	64	12,326	25	18,771	36	15,667	31	16,023	30	34,299	69	36,612	70
*H. tarandi*	4895	13	7219	21.3	3291	26.7	6842	36.4	2997	19.1	3370	21.0	5189	15.1	10,691	29.2
Acute trauma	751	2.0	904	2.7	534	4.3	559	3.0	273	1.7	277	1.7	1012	3.0	1186	3.2
Chronic trauma	145	0.4	328	1.0	144	1.2	162	0.9	59	0.4	419	2.6	230	0.7	71	0.2
IP	536	1.4	1142	3.4	158	1.3	1124	6.0	178	1.1	510	3.2	516	1.5	1756	4.8

The IRR values with 95% confidence intervals (CI) for the total number of conditions found in PMI of reindeer are presented in Table 4. Three variables (year, slaughter season and slaughterhouse size) showed statistically significant differences in the model.

**Table 4.** Incidence risk ratios (IRR) and 95% confidence intervals (CI) of total number of conditions found in PMI of reindeer. Inspectors are divided in two groups with long (LE, > 3 years and > 10,000 reindeer) and short (SE, < 3 years and < 10,000 reindeer) experience in PMI of reindeer.

Characteristics		IRR	95% CI	*p*-value
Year	2015	Reference		
	2016	1.3	1.2–1.5	<0.001
Origin of reindeer	Forest	Reference		
	Mountain	1.0	0.8–1.2	0.861
Inspector experience group	LE	Reference		
	SE	0.9	0.8–1.1	0.300
Slaughter season	Autumn	Reference		
	Winter	1.6	1.4–1.9	<0.001
	Spring	1.3	1.0–1.7	0.018
Slaughter house size	Large	Reference		
	Small	1.9	1.7–2.2	<0.001

Results for *H. tarandi*, acute trauma, chronic trauma and inflammatory processes are presented in Table 5. *Hypoderma tarandi* showed highly significant differences for all covariates/factors tested except inspector experience group. Both acute and chronic trauma showed higher IRR for reindeer of mountain origin and winter season. In small slaughterhouses, IRR was higher for acute trauma and lower for chronic trauma compared with large slaughterhouses. Reindeer were at higher risk of chronic trauma and inflammatory processes (IP) in 2016 compared with 2015 (Table 5). The risks of IP were lower in the spring season compared with winter and autumn, for reindeer originating from mountain areas and for short experience of inspectors (SE). The most frequent IP codes were pneumonia, pleuritis and peritonitis, which together comprised 87% and 84% of all inflammatory processes recorded in 2015 and 2016, respectively.

**Table 5.** Incidence risk ratio (IRR) with 95% confidence intervals (CI) of number of *Hypoderma tarandi*, acute trauma, chronic trauma and inflammatory processes (IP) found in PMI of reindeer. Inspector experience groups are with long (LE) and short (SE) experience in PMI of reindeer.​

Characteristics		*H. tarandi* IRR (95% CI)	*p*-value	Acute trauma IRR (95% CI)	*p*-value	Chronic trauma IRR (95% CI)	*p*-value	IP IRR (95%CI)	*p*-value
Year	2015	Reference							
	2016	1.8 (1.4–2.4)	<0.001	1.1 (0.9–1.3)	0.275	1.6 (1.3–2.3)	<0.001	2.8 (2.2–3.4)	<0.001
Origin of reindeer	Forest	Reference							
	Mountain	2.5 (1.8–3.4)	<0.001	1.8 (1.4–2.3)	<0.001	1.8 (1.2–2.7)	0.004	0.8 (0.6–1.0)	0.051
Inspector experience group	LE	Reference							
	SE	0.9 (0.6–1.2)	0.399	0.9 (0.7–1.2)	0.678	0.8 (0.5–1.3)	0.380	0.7 (0.5–1.0)	0.034
Slaughter season	Autumn	Reference							
	Winter	15.2 (10.1–22.5)	<0.001	1.4 (1.1–1.8)	0.008	1.9 (1.2–2.8)	0.002	1.1 (0.8–1.4)	0.595
	Spring	14.3 (8.6–24.1)	<0.001	1.1 (0.8–1.6)	0.489	1.2 (0.7–2.2)	0.446	0.6 (0.4–0.8)	0.004
Slaughter house size	Large	Reference							
	Small	2.0 (1.5–2.8)	<0.001	0.4 (0.3–0.7)	<0.001	1.9 (1.3–2.8)	<0.001	0.8 (0.5–1.3)	0.300
Inspector experience group* slaughterhouse size	Group SE* Small	No interaction	-	4.6 (2.8–7.6)	<0.001	0.4 (0.2–0.9)	0.023	2.1 (1.3–3.7)	0.004

*Interaction between inspector experience group SE and small slaughter houses is marked with “SE * Small”.

No interaction between slaughterhouse size ‘small’ and inspector experience group ‘SE’ was found in general data or for *H. tarandi*. However, a significant interaction was found in the combined data for acute trauma, chronic trauma and IP (Table 5). Analysis of both years separately showed an IRR for the interaction with acute trauma in 2015 of 2.3 (CI 1.1–5.0, *p *= 0.029), while in 2016 IRR was 8.5 (CI 4.3–16.8, *p *< 0.001). For chronic trauma, this interaction was seen only in 2016 (IRR 0.3, C. I. 0.1–0.95, *p *= 0.028), as was also the case for IP (IRR 2.7, CI 1.3–5.7, *p *= 0.006).

## Discussion

Reindeer herding is the legally protected traditional livelihood for the Sami minority in Sweden. The management strategy used by herders is extensive and the reindeer population can be considered more wild than domesticated. Health problems perceived by herders relate to nutrition and calving in fenced enclosures. Production of meat (1375 t, 5.5 kg/live reindeer in winter herd) is low compared with in Finland (1980 t in 2014/2015, 10.3 kg/live reindeer in winter herd [9]), where reindeer management is often part of more intensive agricultural small enterprises. Reindeer losses caused by uncontrolled external circumstances are detrimental for management in Sweden,[10] as large predators alone can cause a 52–63% reduction in annual carcass production.[11] The number of reindeer slaughtered per year in Sweden is about 20% of the whole population. Almost all reindeer are slaughtered in slaughterhouses and thus presented for official MI. Slaughtered reindeer are almost exclusively from the Swedish reindeer population and only 2% of the population goes to slaughter in Finland. The proportions of slaughtered reindeer reared in mountain and forest environments reflect the proportions of the living reindeer population. Hence the results presented here are representative of the Swedish reindeer population at slaughter. We concluded that PMI can be a helpful tool for monitoring different hazards among reindeer.

### Carcass conditions found in PMI

In general, more carcass conditions were found in 2016 than in 2015. This pattern was seen even for *H. tarandi*, chronic trauma and IP incidence. Possible causes of this difference should be investigated further by analysing all conditions on code level within and between the study years. The possibility of misclassifications contributing to biased results cannot be excluded. However, since 2015 internal calibration of PMI in reindeer slaughter has involved compulsory regular quarterly meetings and individual supervision activities for all staff in PMI of reindeer. Before 2015, intercalibration activities comprised the steering documents available and compulsory training for PMI staff in reindeer slaughter every second year.

The probability of detecting carcass conditions in small slaughterhouses compared with large was about two-fold higher for *H. tarandi* and chronic trauma. This was unexpected, because of the uniform geographical distribution of small and large slaughterhouses throughout the reindeer herding areas. Origin (mountain/forest) of the reindeer slaughtered was also evenly distributed between large and small slaughterhouses.

There was no interaction between inspector experience group and slaughterhouse size for total number of PMI findings or for *H. tarandi* incidence. However, a significant interaction was found for acute and chronic trauma and IP. Further analysis showed that the interaction was strongest for acute trauma, while for IP and chronic trauma an interaction emerged only in 2016. The findings with regard to inspector experience were surprising and will require further study (Tables 2 and 5). For example, the effects of variation in experience, type of slaughterhouse, slaughter speed, inspector vigilance and the current internal training system have to be further evaluated.

The fact that more carcass conditions were recorded in winter and spring is interesting (Table 4). This can probably be at least partly explained by biological factors, for example more apparent parasites or more advanced emaciation because of natural catabolism. Even management-related factors such as transportation, absence of supplementary feeding in areas with bad pasture or handling of animals can result in more pronounced findings at later slaughter.

### Parasites

The parasites recorded were different kinds of non-zoonotic parasites that are considered part of the reindeer ecosystem. The most abundant parasites found were subcutaneous larval stages of *H. tarandi*. Within-batch prevalence varied greatly in both years. This should be studied further in order to detect possible changes in parasite population dynamics and links to different reindeer management routines in different ecological contexts. The PMI results on parasites could be valuable feedback for reindeer owners seeking to optimise their management, e.g. antiparasite treatment strategies.

*Hypoderma tarandi* was recorded 2.5-fold more frequently in reindeer of mountain origin than in forest reindeer. This was expected, since high latitude and open landscape are reported to be some of the risk factors for *H*. *tarandi*.[12] In winter and spring slaughter, *H. tarandi* was found 14–15-fold more frequently than in autumn slaughter. As larval stages grow, they become more visible with their subcutaneous capsule during winter. Consequently, during autumn there are fewer findings of this parasite as the larvae are less visible early in their biological development cycle.

In this study, *Setaria* sp. was found in low numbers, mainly in slaughter batches from the Torne valley, close to the Finnish border. The prevalence of this particular mosquito-borne filaroid nematode is interesting because of the potential increase in this species due to possible warmer summer mean temperatures in reindeer herding areas.[13] It appears that interactions among mammals, arthropods and parasites are complex and a mean summer temperature exceeding 14°C could drive the emergence of disease caused by *Setaria tundra*.

### Inflammatory processes

The IP diagnostic group consists of a variety of inflammations mainly relating to the serosa lining of the thoracic and abdominal cavity. Aetiology for IP can be parasite-related but is not coded as such, because of absence of larvae or pathognomonic signs. This perhaps explains the steep decrease in the IRR value for IP towards spring compared with winter, since as parasites grow they can be diagnosed more easily. The need for longer experience to recognise IP can explain the lower IRR for less experienced staff in general. The interaction between small slaughterhouses and less experienced inspectors, which resulted in higher IRR for IP in 2016, can be explained by lower slaughter speeds and even by internal training in 2015. One future research question is the possibility of reindeer with IP having co-morbidities, i.e. a higher risk of trauma injuries. In addition, peritonitis in particular should be analysed further in coming years, in order to see if there is an increase related to *Setaria* sp. similar to that observed in Finland.[14]

### Zoonotic hazards

The only macroscopic parasite of public health interest in reindeer diagnosed in Sweden, *Echinococcus granulosus* (hydatid tapeworm), was not found in this study. However, according to national zoonosis surveillance, *Echinococcus* sp. prevalence in reindeer slaughtered in northernmost Sweden in 1973, before compulsory MI, was about 2%. The most recent cases were diagnosed during winter 1996/1997.[15] Numbers of the crucial host of the synanthropic cycle of *Echinococcus* sp., namely reindeer-herding dogs,[16] are now low and those in use are regularly treated by their owners with anthelmintics.

Bovine tuberculosis (*Mycobacterium bovis*), a harmful disease in both humans and reindeer, was not seen at all in this study. The majority of reindeer slaughtered in Sweden are calves under one year old and therefore it is less likely that PMI will detect conditions like bovine TB because the clinical and/or pathological signs in affected animals will not yet have developed. Consequently, complementary information on the presence of *Mycobacterium bovis* in reindeer is needed from other sources. In national surveillance of wild predators of reindeer and other animals, *Mycobacterium bovis* has not been found in Sweden.[17] Moreover, *Mycobacterium bovis* has not been detected in pre-export testing and routine necropsies of reindeer (Kautto et al., unpublished data). *Mycobacterium bovis* is not observed in reindeer in Finland [18] and Norway,[19] which minimises the risk of introduction by animals naturally crossing Sweden’s borders. Hence, the claim of TB freedom in reindeer appears to be true in Nordic reindeer herding areas.

Consequently, visual-only PMI of reindeer could be a preferred procedure from a hygiene perspective. On the other hand, elimination of routine palpation and incision is detrimental for the ability to detect tuberculosis in general in PMI.[20] However, as TB freedom in reindeer appears to be true, continued PMI with routine palpation and incisions would not lower the TB risk. Moreover, the already ongoing integrated monitoring of wildlife, including predators of reindeer, can detect any emergence of bovine TB in reindeer.

Slaughter hygiene was good in this study, which reflects the professionalism of food business operators. This is important because reindeer can be carriers of bacterial foodborne pathogens.[21] Moreover, the high status and price of reindeer meat compared with beef, mutton or pork encourages good manufacturing practices at slaughter.

### Conditions related to animal welfare

Trauma injuries were the third most common condition found in PMI. The total prevalence of all trauma injuries at slaughter was 3–4% for 2015–2016. This included old fractures, mainly of the ribs, and wounds, as well as more acute injuries such as subcutaneous haemorrhages. Causes of these trauma injuries include predators and rutting fights between bulls, but also road or railroad accidents. In addition, there were management-related trauma injuries caused during gathering, transport and slaughter. Reindeer are handled very few times a year and the degree of domestication is low. Even if handling is done carefully, reindeer are brought together during these activities and it is natural for flight behaviour and acts of hierarchy such as butting, kicking and riding on the back to occur. Acute subcutaneous bruises are the most common condition in such cases.

The individual reindeer prevalence of acute trauma injuries was below 3% in 2015–2016, but in all batches one or more reindeer had acute trauma injuries, i.e. batch prevalence was quite high. Chronic trauma had both lower individual and batch prevalence. Some of the reindeer with injuries die in the wilderness, which may lower the observed prevalence of chronic trauma at slaughter. On the slaughter line, acute trauma can even mask chronic trauma. The level of domestication varies greatly between different Sami villages, being lowest in mountain villages. This can explain the 1.8-fold higher risk of both acute and chronic trauma injuries found for mountain compared with forest reindeer (Table 5). Gatherings with large numbers of reindeer handled at the same time are also more common in mountain villages and especially affect young animals.

Emaciation was recorded in low numbers, but was still one of the most common conditions found in PMI. Reindeer are under physiological catabolism during winter, which can cause severe problems for old reindeer with worn teeth and for calves in which the rumen villi are not yet fully developed. These animals are most likely to suffer during harsh winters and on bad pasture. Fecundity is also likely to be affected.[22,23] Under the extensive reindeer management system in which the vast majority of reindeer are herded in Sweden, supplementary feeding is used only in severe cases with totally blocked pasture and does not always reach all reindeer widely spread over the terrain. To avoid loss of reindeer, corrals can be set up. This management strategy has a longer tradition in Finland [24] and can be one of the reasons for the difference in reindeer meat production efficiency between the two countries. Emaciated carcasses can have traces of parasite infestation and indications of systemic illness. The host–parasite dynamics [25] need to be analysed in every subpopulation of reindeer before conclusions are drawn concerning the causal effect of parasites on the health status of reindeer.

### Environmental conditions

The number of reindeer recorded as having excessively high levels of caesium at slaughter is close to negligible today, but was high in the past following the Chernobyl nuclear accident in Ukraine in April 1986, which was followed by radioactive fallout in some areas of Sweden. Official surveillance of caesium in reindeer has been performed since 1986. The half-life of Cs^137^ is about 30 years and living reindeer with high levels of caesium are now rare. The national surveillance programme for residuals of veterinary medicines, other undesirable substances and pollutants in the food chain according to EU Directive 96/23/EG [26] and national legislation [27] also show low levels in reindeer.[6] Consequently, reindeer meat can be considered safe food in terms of chemical hazards today. However, further national surveillance is still demanded by legislation and is justified by the possible effects of climate change on the environmental distribution and toxicity of chemical pollutants affecting sensitive sub-arctic ecosystems.[28] Sampling in reindeer slaughterhouses can use reindeer as an efficient sentinel in that sense.

### Post mortem meat inspection as a monitoring tool

To our knowledge, there are no studies or data available concerning the sensitivity and specificity of PMI at reindeer slaughter. Conventional PMI on pigs can lack sensitivity and therefore underestimates the prevalence of some conditions and diseases.[29] However, PMI is a suitable source of data for domesticated animals,[30] semi-domesticated reindeer included,[31] because when many relevant food safety and animal welfare conditions have low prevalence at population level, PMI gains strength through the large number of animals inspected, and thereby monitored. Care should be exercised when interpreting the results, as misclassification biases must be expected. However, we would argue that these misclassifications are non-differential if the following caveats are observed. By focusing on the numbers relating to the diagnostic groups (Table 1), the comparisons should be more robust. Moreover, for comparisons between years or between slaughterhouses, then the intercalibration and understanding of diagnostic criteria between veterinary inspectors must be assumed to be equivalent.

Nevertheless, the reindeer population resembles that of wild ungulates and fallen stock numbers include individuals dying in the wilderness. Not capturing fallen stock represents a selection bias as regards the whole reindeer population, but PMI still captures food safety risks that might enter the food chain through reindeer meat. Hence, when using PMI findings on reindeer as part of the environmental monitoring system, the relevance and possible bias of each indicator or metric used need to be carefully assessed. One argument for using PMI is that reindeer range freely on pastures and veterinary interventions are extremely rare, so veterinary treatment or autopsy records are not a monitoring alternative.

The current conventional PMI procedure for reindeer includes routine palpation and incision. Histological and serological samples are taken with some conditions, in order to confirm the diagnosis. Food chain information drives caesium sampling, i.e. it is only done on reindeer from risk pastures. The European Food Safety Authority (EFSA) lists *Salmonella* sp. and *Toxoplasma gondii* as the most relevant hazards to be covered by MI of farmed game from a public health perspective.[20] Neither these nor the recently emerged chronic wasting disease in Norway [32] can be detected in domesticated or game animals by conventional macroscopic PMI in slaughterhouses. Hence, PMI should be complemented with blood and/or tissue samples, preferably taken at slaughter, to monitor these pathogens and other hazards of importance.

Furthermore, visual-only PMI of farmed and wild deer has a negligible negative effect on the identification of hazards relevant for public health and animal welfare.[30] When prevalences of some findings are low, it is highly unlikely that a decline in animal-level sensitivity would significantly impact herd-level sensitivity in these cases.[33] We suggest that the same applies to reindeer.

The majority of slaughtered reindeer are calves, which are in fact the stratum in the population that is most sensitive to circumstances causing animal welfare problems (bad pastures, harsh handling, etc.). Hence, PMI focuses on high risk groups and PMI of reindeer provides good epidemiological indicators of animal welfare problems. Visual PMI is a reliable method for monitoring both trauma injuries and emaciation in all reindeer slaughtered.

## Conclusions

Official inspections at reindeer slaughter cover the majority (98%) of reindeer slaughtered every year and are the best monitoring system available for detecting food safety and animal welfare problems, zoonoses and notifiable diseases in the reindeer population in Sweden.

The conditions recorded in PMI of reindeer rarely indicate public health hazards and no epizooties or zoonoses have been recorded in the past 20 years. Available PMI data support the claim that the reindeer population in Sweden is TB-free.

The inspection data reflect the health and welfare status of the reindeer population and risks to food safety and the environment in Sweden. Some findings in this study, e.g. an interaction between inspector experience and slaughterhouse size, are interesting and should be studied further.

Fully efficient, risk-focused meat inspection of reindeer can be achieved by visual PMI of reindeer, with complementary blood and/or tissue sampling focused on specific hazards when required. This would be a suitable method for monitoring the health and welfare of the reindeer population, the food safety risks entering the food chain with reindeer meat and the status of the environment.
